# Men’s utilisation of sexual and reproductive health services in low- and middle-income countries: A narrative review

**DOI:** 10.4102/sajid.v38i1.473

**Published:** 2023-06-20

**Authors:** Mpumelelo Nyalela, Thembelihle Dlungwane

**Affiliations:** 1 School of Nursing and Public Health, College of Health Sciences, University of KwaZulu-Natal, Durban, South Africa

**Keywords:** men, sexual and reproductive health services, factors influencing SRH service utilisation, factors facilitating or inhibiting the SRH service utilisation, low- and middle-income countries

## Abstract

**Background:**

Men have poor access to sexual and reproductive health (SRH) services globally, particularly in low- and middle-income countries (LMICs). Nevertheless, in LMIC and high-income countries (HICs), low SRH utilisation happens on account of several factors, such as individual, health system-related, and sociocultural factors. Identifying and addressing men’s SRH service underutilisation remains essential to improving their sexual health and averting higher mortality and early morbidity associated with poor health seeking behaviour (HSB) among men.

**Aim:**

This narrative review identifies factors influencing whether men do or do not utilise SRH services in LMICs.

**Setting:**

We report on articles published in LMICs: Africa, Asia and South America.

**Method:**

In this narrative review, we searched for quantitative and qualitative articles published between 2004 and 2021 from international databases, including Google Scholar, ScienceDirect, EBSCOhost, Scopus, PubMed, Medline, and reference lists of retrieved published articles.

**Results:**

A total of 2219 articles were retrieved, from which 36 met the inclusion criteria. Factors contributing to poor uptake of SRH services by men included: a lack of access and availability of SRH services, poor health-seeking behaviour among men, and SRH facilities not being perceived as ‘male-friendly spaces’. Furthermore, our review reveals that decreased use of SRH services is attributed to factors such as a lack of focus on men’s SRH.

**Conclusion:**

The current underutilised state of SRH services calls for urgent implementation of evidence-based interventions. Identifying men’s SRH service inhibitors and enablers will assist programme managers and policymakers in designing SRH services tailored to their sexual health needs.

**Contribution:**

Despite numerous global interventions to motivate men, the findings provide insight into the underutilisation of SRH services. The study also reveals the inadequate comprehensive investigation of men’s SRH service utilisation, especially older men, to comprehend men’s problems fully. Further research needs to be conducted on SRH issues, including vasectomy, mental health, and chronic conditions related to sexual and reproductive health. The analysis can assist SRH policymakers and program managers in strengthening the policies to motivate men to engage better with SRH services.

## Introduction

Sexual and reproductive health (SRH) is a global public health concern.^1,2^ Sexual and reproductive health problems account for major health challenges and constitute almost 14% of the disease burden, contributing to higher mortality and earlier morbidity in men.^3^ Globally, SRH is a fundamental human right of every individual,^1^ but only less than one-quarter of men report utilising SRH services.^4^

The utilisation of SRH services refers to having timely and convenient access to these services.^5,6^ The services provide a state of physical, mental, and emotional well-being related to sexuality and reproduction, and are essential for the socioeconomic development of communities and countries.^2,7,8,9^ Sexual and reproductive health services include: contraception, prevention and treatment of mental disorders, communicable and non-communicable diseases, male medical circumcision (MMC), and psychosocial interventions such as sexual health counselling.^5,9,10,11,12,13^

Despite men’s need for SRH services, most studies report underutilisation of SRH by men.^4,10,14,15^ This is evident despite several international conventions, adopted programmes and policies that seek to educate men and boys for reproductive health services.^10,12^ The Guttmacher–Lancet Commission (2018) posited that 4.3 billion people of reproductive age would inadequately utilise SRH services throughout their lives.^2^ Factors influencing men’s underutilisation are multifaceted. These factors include the lack of focus on men’s SRH by international programmes such as Sustainable Development Goals (SDGs) and Family Planning 2020 (FP2020), which largely focus on women and youth.^13,14,15,16^ Furthermore, SRH service provision is often fragmented or poorly structured for men’s health needs and is mostly needlessly expensive.^3,16^

The purpose of the review is to synthesise available evidence on factors influencing whether men do or do not utilise SRH services in low- and middle-income countries (LMICs). The review focused on LMICs because of their resource constraints which lead to poorer access and a higher unmet need for SRH services. This review aimed to answer our research question: ‘What factors influence whether men do or do not utilise SRH services in LMICs?’

## Methodology

The non-systematic narrative literature review method was followed to identify relevant literature. The search included published peer-reviewed articles, reports, and grey literature. Peer-reviewed articles were searched in the following search engines and databases: Google Scholar, ScienceDirect, Scopus, and EBSCOhost. Additionally, electronic databases: CINHAL, PubMed, Medline, Academic Search Complete, Health Source – Consumer Edition, Health Source: Nursing/Academic Edition, and MEDLINE, electronic journals, and reference lists of retrieved published articles were also searched.

The following Medical Subject Headings (MeSH) terms were used to retrieve relevant articles for this review: ‘sexual and reproductive health services,’ ‘men,’ ‘factors influencing or inhibiting/facilitating SRH service utilisation,’ and the ‘utilisation of SRH services by men in LMICs’. We also used keywords from the extracted articles to help narrow the focus. To cover SRH services comprehensively, studies were also searched based on individual SRH services, for example, using condoms, vasectomy services, HIV services, prostate cancer (PC), family planning (FP), MMC, and infertility. Studies that investigated Health Care Workers’ (HCW) knowledge and perceptions about men’s use of SRH services were also included.

This review included quantitative and qualitative papers published in English between 2004 and 2021. Studies conducted in 2004 were sourced because the World Health Organization (WHO) first recognised the immense global health burden associated with poor quality of care for people’s SRH in 2004.^6^ The database was constructed to extract important dimensions of factors influencing SRH service utilisation. We extracted only relevant results about populations eligible for inclusion. For example, where data were collected from males and females, we extracted only data presented for males. Hence, studies focused exclusively on women were excluded.

We included studies reporting on factors influencing SRH service utilisation by men aged 15 years and above. We aimed to target studies focusing on older men because the literature review indicated less focus on SRH service utilisation among this population. Therefore, studies reporting exclusively on the factors influencing adolescent SRH service utilisation were also excluded. However, studies including subjects aged 15 years and above were included because of overlapping reporting on the studies focusing on this review’s research question and the paucity of studies focusing exclusively on older men. For example, identified studies focused on ages 15 years and above, 15–25 years, or 15–69 years. The research focusing on SRH service utilisation among adolescents is over-researched, as approximately 90% of studies identified in this review focused exclusively on adolescents. Studies reporting on the interventions and the evaluation of interventions to improve SRH utilisation, clinical decision-making algorithms, studies conducted in high-income countries (HICs), and studies not reporting on barriers or facilitators of SRH utilisation were also not considered.

The database search generated 2219 records, including grey literature, health services reports, original research articles, theses, and dissertations. Full-text articles were derived from different databases and analysed separately by two researchers. After that, the researchers agreed by collating all relevant articles for the study. On a review of titles, 2042 were excluded because of duplication, unrelated to this narrative review topic, or exclusively focused on adolescents and women. On a review of abstracts, 96 papers were excluded because they reported on the evaluation of SRH services, management of SRH problems, or male involvement in partner FP. These policies and documents did not report on factors facilitating or hindering SRH service utilisation. The latter were systematic reviews.

**FIGURE 1:**
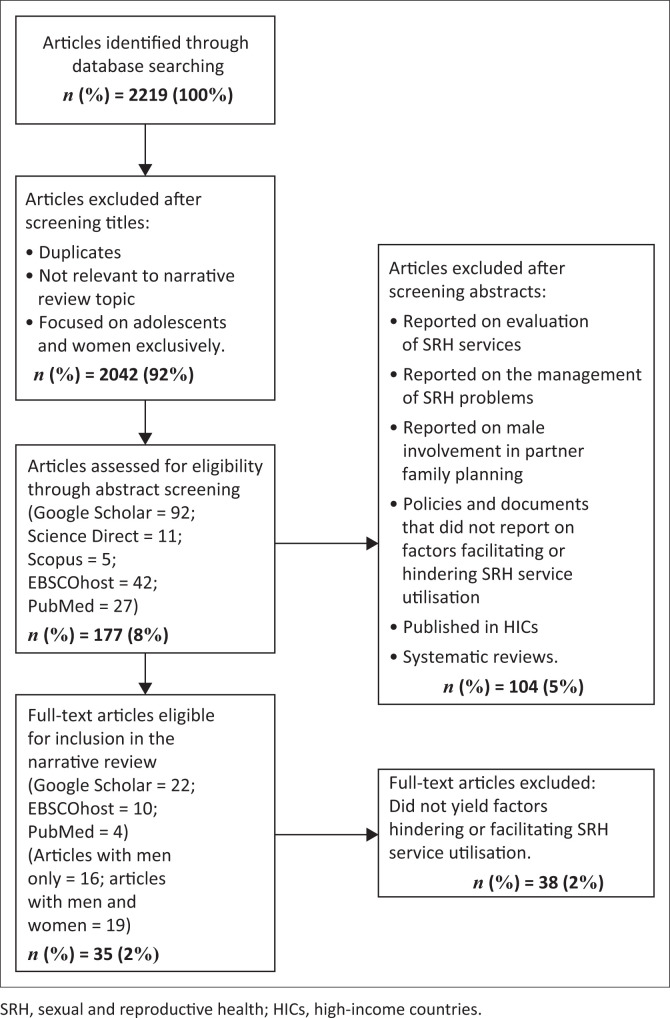
Flow chart mapping out the number of articles identified, screened, and excluded together with reasons for exclusion.

Of 73 potentially relevant full-text papers, 37 were excluded because the results did not yield factors hindering or facilitating SRH service utilisation. Thirty-six papers met the inclusion criteria and were captured in the database. Secondary to the heterogeneity of papers, we present a narrative synthesis describing study characteristics and key findings. We further summarise overarching themes and the consistency of key findings. A database was constructed to summarise the studies identified for the review. The following information was captured in the database: (Table 1) – author(s), year of publication, the country(s) where the study was conducted, participant characteristics (age and gender), the study design, study setting (urban or rural); and (Table 2) – author(s), barriers, facilitators, and SRH issues. Figure 1 presents the search algorithm indicating the number of identified studies, included and excluded studies, and reasons for exclusion.

**TABLE 1:** Summary of study characteristics that met inclusion criteria.

Authors	Year	Study location	Participant characteristics (age and gender)	Study design	Study setting (urban or rural)
Hoffman et al.	2015	South Africa	Men and women 18 years and above	Mixed-analytical cross-sectional study design	Urban
Humphries et al.	2015	South Africa	Men 18–54 years	Qualitative – FGDs	Rural
Zissette et al.	2016	South Africa	Men 24–80 years	Qualitative – IDIs	Urban
Khan et al.	2014	South Africa	Men 18–50 years	Survey questionnaire	Urban
Mwisongo et al.	2016	South Africa	Men and women 16 years and above	Quantitative – cross-sectional survey (semi-structured questionnaire)	Urban and rural
Stern et al.	2014	South Africa	Men 18–55 years	Qualitative – FGDs	Urban and rural
Morison et al.	2016	South Africa	Men and women 20–90 years	Quantitative – Survey – A healthcare user questionnaire	Urban and rural
Chikovore et al.	2016	South Africa	Men 17–64 years	Qualitative – IDIs, FGDs	Rural
Evens et al.	2014	Kenya	Men and women 18–35 years	Qualitative – FGDs, IDIs	Urban and rural
Herman-Roloff et al.	2011	Kenya	Men 18–40 years	Qualitative – FGDs	Urban and rural
Withers et al.	2015	Kenya	Men 15–64 years	Quantitative – Kenya demographic and health survey (KDHS).	Urban and rural
Godia et al.	2013	Kenya	Men and women 27–50 years	Qualitative – FGDs, IDIs	Urban and rural
Ssekubugu et al.	2013	Uganda	Men 15–49 years	Quantitative – RCT qualitative – FGDs	Rural
Nalwadda et al.	2010	Uganda	Men and women 15–25 years	Qualitative – FGDs	Urban and rural
Sunnu et al.	2016	Ghana	Men and women 15–65 years	A quantitative – cross-sectional survey	Urban
Leblanc et al.	2015	Ghana	Men 18 years and above	Mixed: Quantitative – self-administered structured questionnaire. Qualitative – IDIs, FGDs	Urban
Adongo et al.	2014	Ghana	Men and women 18 years and above	Qualitative – IDIs, FGDs	Urban and rural
Hatzold et al.	2014	Zimbabwe	Men 15–49 years	Mixed: Quantitative – National, population-based survey, qualitative – FGDs	Urban and rural
Skovdal et al.	2011	Zimbabwe	Men and women 18 years and above	Qualitative - IDIs, FGDs	Rural
Thomas et al.	2015	Nigeria	Men and women 18–30 years	Quantitative – interview schedule – pre-tested questionnaire (scale)	Rural
Jones et al.	2017	Nigeria	Men and women 18–24 years	Qualitative – IDIs	Rural
Hassan et al.	2015	Nigeria	Men 25–60 years	Quantitative – descriptive cross-sectional	Urban
Tamang et al.	2017	Nepal	Men and women 15–24 years	The quantitative – cross-sectional household survey	Urban
Gautam et al.	2018	Nepal	Men and women 15–24 years	Qualitative – IDIs	Rural
Regmi et al.	2010	Nepal	Men and women 18–22 years	Qualitative – IDIs and FGDs	Urban and rural
Shattuck et al.	2014	Rwanda	Men and women 24–45 years	Quantitative – cross-sectional descriptive	Urban and rural
Adams et al.	2015	Swaziland	Men 18–49 years	Qualitative – FGDs, IDIs, participant observation	Urban and rural
Kelly et al.	2012	Papua New Guinea	Men 16 years and above	Qualitative – FGDs, IDIs	Urban and rural
Skolnik et al.	2014	Lesotho	Men 18 years and above	Mixed: Quantitative – cross-sectional Qualitative – FGDs	Urban and rural
Yabeny et al.	2018	Mexico	Men 20–39 years	Qualitative – IDIs	Urban
Parcon et al.	2010	Philippines (Western Visayas)	Men 15–54 years	Quantitative – National Health and Demographic Survey (NDHS)	Urban and rural
Muntean et al.	2015	Ethiopia	Men and women 15–24 years	Qualitative – IDIs	Urban and rural
Oraby et al.	2013	Egypt	Men and women 15–24 years	Qualitative – IDIs, FGDs	Urban and rural
Thongmixay et al.	2019	Lao	Men and women 15–25 years	Qualitative – IDIs	Urban and rural
Zaw et al.	2012	Myanmar	Men and women 15–24 years	Quantitative – cross-sectional study	Urban and rural

FGD, focus group discussions; IDIs, in-depth interviews; RCT, randomised control trial.

**TABLE 2:** Summary of factors influencing sexual and reproductive health services.

Authors	Barriers	Facilitators	SRH issues
Hoffman et al.	Intra- and postprocedure complications Low-risk perception, Lack of social support High costs Fear HIV test	Protection against diseases (HIV, STIs, cancers) acquisition Hygiene Virility Good societal standing	MMC
Humphries et al.	Postprocedure complications Low-risk perception	Virility Social support	MMC
Zissette et al.	Threat to masculinity (stigma around HIV) Fear of losing manhood	Social support Good standing example	HIV testing and management
Khan et al.	Lack of confidentiality High costs		HIV counselling and testing (HCT)
Mwisongo et al.	Fear of HIV test (stigma around HIV) Staff attitudes Lack of knowledge (testing sites, understanding HIV) Inconvenient opening hours Fear of death Ignorance (lack of condom use) Low-risk perception High costs (traveling) Lack of social support	Incentives for those who test for HIV Good staff attitudes Role modes Community testing	HIV testing
Stern et al.	Condom use – virility (sex interruption, uncontrollable sexual urge) Lack of knowledge of HIV transmission Substance abuse Low-risk perception	None	Condom use
Morison et al.	Long waiting times Unavailability of medicines and equipment Staff attitudes Lack of privacy and confidentiality High costs (traveling)	None	HCT Family Planning Condoms use HIV and STI treatment and counselling
Chikovore et al.	Fear HIV test low-risk perception Preference for traditional medicine	None	HCT and management
Evens et al.	Myths (circumcised penis would tear a condom) Nonculture, nonreligion Lack of trust in government witchcrafts beliefs Low-risk perception Fear HIV test	Virility	MMC
Herman-Roloff et al.	Fear of losing a job Noncultural, nonreligious Intra- and postprocedure complications Lack of knowledge Vulnerability to ignorance Distance to health facilities Female service providers	Hygiene Social acceptance Virility Protection against diseases Convenience (easier to use a condom)	MMC
Withers et al.	Lack of knowledge or awareness Religious prohibition Unvirility Hindrance to community development	None	Family planning
Godia et al.	Limited knowledge and competency of HSP Staff attitudes Lack of medicines and equipment Lack of confidentiality and privacy Long waiting times High costs Inconvenient hours	None	Family Planning STI/HIV services Condom use
Ssekubugu et al.	Postprocedure complications Fear HIV test Myths (infertility) Partial protection against diseases Long waiting times Fear of losing a job	Protection against diseases Hygiene Social support Incentives	MMC
Nalwadda et al.	Myths (infertility, porous and infectious condoms), virility Low-risk perception Incontrollable sexual urge Staff attitudes Lack of privacy and confidentiality Lack of medicines and equipment High costs Long distance to health facilities Inconvenient opening hours Long waiting times	None	Family Planning STI/HIV services Condom use
Sunnu et al.	Lack social support Nonreligious, noncultural Staff attitudes Distance to health facilities	None	Family planning
Leblanc et al.	Cultural beliefs (non-condom use) Embarrassment Desire for children Fear of HIV test (stigma) High costs Lack of confidentiality	None	HIV services Condom use
Adongo et al.	Lack of social support Unvirility Vulnerability to ignorance Intra- and postprocedure complications Lack of knowledge Religious doctrines	None	Vasectomy Condom use
Hatzold et al.	Intraprocedure complications Low-risk perception Lack of social support High costs Fear of an HIV test Myths (infertility)	Protection against diseases Hygiene Virility Set a good example	MMC
Skovdal et al.	Fear of HIV test Stigma Embarrassment Threat to masculinity Low-risk perception Lack of knowledge	Social support Role models	HIV services
Thomas et al.	Fear of HIV test Stigma Nonculture	None	HIV services
Jones et al.	Stigma Inaccessibility Lack of knowledge Inaccessibility Low-risk perception	None	HIV services
Hassan et al.	Low-risk perception Lack of time Intraprocedure complications Fear of PC test outcome Lack of knowledge	None	Prostate Cancer screening
Tamang et al.	Embarrassment Poor health services Lack of knowledge Inaccessibility	None	Condom use Family planning
Gautam et al.	Embarrassment Lack of knowledge Staff attitudes Lack of privacy and confidentiality Lack of medicines and equipment	None	All
Regmi et al.	Embarrassment Poor health services Lack of knowledge Substance abuse Inaccessibility High costs	None	Condom use
Shattuck et al.	None	Financial relief (limit family size) Complications of other family planning methods Permanent method Low-risk of complications	Vasectomy services
Adams et al.	Threat to masculinity Virility Intra- and postprocedure complications Partially protective against diseases Lack of trust in government Noncultural, nonreligious Lack of knowledge Myths (foreskins used for witchcraft)	Virility Protection against diseases Convenience (wearing condoms)	MMC
Kelly et al.	Vulnerable to ignorance Noncultural, nonreligious	Protection against diseases Hygiene Culturally appropriate Virility	MMC
Skolnik et al.	Intraprocedure complications Fear of HIV test High costs Female health workers Long waiting times	Protection against diseases Hygiene Social support Virility	MMC
Yabeny et al.	Low-risk perception	Fear of illness Mistrust in relationship	Condom use
Parcon et al.	Lack of knowledge Myths (loss of libido) Unvirility decreased, sexual activity, loss of vitality Inconvenience Embarrassment	Protection against diseases	Vasectomy Condom use
Muntean et al.	High costs Long distance to the health facility Staff attitudes Inconvenient location of facilities Inconvenient hours Lack of privacy and confidentiality Embarrassment Lack of knowledge Noncultural	None	All
Oraby et al.	Inaccessibility Absence of male health workers	None	All
Thongmixay et al.	Lack of medicines and equipment Low-risk perception Lack of knowledge Lack of privacy and confidentiality Substance abuse Noncultural, nonreligious Embarrassment High costs	Protection against diseases Social acceptance	Condom use Family planning
Zaw et al.	High costs Lack of confidentiality Lack of transport Staff attitudes Fear of HIV test Embarrassment Lack of knowledge	None	Family planning HIV services Condom use

SRH, sexual and reproductive health; VMMC, voluntary male medical circumcision, PC, prostate cancer; STIs, sexually transmitted infections; HSP, health services personnel.

### Ethical considerations

Approval to conduct the study was provided by the Biomedical Research Ethics Committee (BREC) University of KwaZulu-Natal (number: BE 347/19).

## Results

Of 2219 articles retrieved, 36 studies met the inclusion criteria for our narrative literature review. The review includes quantitative, qualitative, and mixed methods studies that reported evidence on factors influencing SRH service utilisation by men. Table 1 presents the author, year of publication and study location, participant characteristics (age and gender), and study design. Table 2 presents barriers, facilitators, and SRH issues. All articles (qualitative and quantitative studies) extracted from the literature were descriptive. The results are summarised narratively.

## Description of included studies

Most studies were conducted in South Africa (22%).^21,22,23,24,25,26,27,28^ Four (11%) studies were conducted in Kenya^29,30,31,32^ three (8%) were conducted in Ghana,^33,34,35^ Nigeria,^36,37,38^ and Nepal^39,40,41^ and two (6%) were conducted in Zimbabwe^42,43^ and Uganda.^44,45^ One study was conducted in Rwanda,^46^ Swaziland,^47^ Papua New Guinea,^48^ Lesotho,^49^ Mexico,^50^ Philippines,^51^ Ethiopia,^52^ Egypt,^53^ Myanmar,^54^ and Lao.^55^ The included studies targeted men aged 15 years and above. Most studies (55%) exclusively targeted men, and 45% focused on both men and women. Eighty-six percent of studies targeted men aged 26 years and above. The remaining 14% targeted men aged between 15 and 25 years.

Most studies that met the inclusion criteria were conducted qualitatively and employed either focus group discussions (FGDs) or in-depth interviews (IDIs), and some both FGDs and IDIs (24%). Twenty-five percent of quantitative studies employed a cross-sectional design, and two employed the National Health and Demographic Survey (NHDS). The latter (16%) used mixed methods. Only one mixed study included randomised control trial (RCT) data. Forty-nine percent of studies were conducted in both urban and rural settings. In these settings, data were collected in towns and townships, rural villages where households were visited, and some participants were recruited on the streets. In studies conducted in public and private-sector health facilities (clinics and hospitals), data were collected in private rooms or offices in community centres, non-governmental organisations (NGOs) sites, and district health offices. The SRH issues identified were vasectomy; FP, MMC; condom use; management and prevention of STIs; HIV services; PC screening, and erectile dysfunction (ED).

### Barriers to men’s utilisation of sexual and reproductive health services

Several barriers have been associated with whether men do or do not utilise SRH services. In the same vein, some studies revealed that, poor health-seeking behaviour could be a barrier to SRH services utilisation.^56,57^ The barriers to men’s SRH service utilisation can be categorised into health service system factors, lack of knowledge, individual and/or personal factors, sociocultural and religious, socioeconomic, and geographical factors, as detailed further.

#### Health service system (physical accessibility, availability, accessibility, affordability)

Amidst health service system factors contributing to underutilising SRH services, inconvenient or limited operating service hours^25,32^; the awkwardness of the location of the SRH services^52^; long-waiting times because of lengthy queues,^27,45^ the fact is that SRH services globally, and especially in LMICs, are largely focused on the needs of women (of reproductive age) and offer little for boys and men.^12,13,15,17^ Further hindering health system factors include poor service quality and lack of materials such as condoms and medicines.^37,40,45^ Men may avoid using SRH services because of hostile and judgemental attitudes from female health service providers, especially towards young and unmarried men,^25,27,32^ and not having same-sex health workers. Some men felt embarrassed to discuss their health issues or be examined by female health workers.^30,49,53^ Notably, most studies that focused on the utilisation of SRH services such as contraceptives, STI and/or HIV services, and condoms and comprehensively on various services revealed a lack of privacy, respect, and potential breaches of confidentiality from the HCW at the health facilities as deterrent to men’s utilising of SRH services.^32,39,45,54,55^

#### Lack of knowledge

Men’s underutilisation of SRH services was associated with a lack of knowledge and awareness of disease such as PC and HIV by SRH screening services.^38,43^ In some studies, a lack of knowledge and understanding of HIV was evident when men often inferred their status from their female partners’ results.^23,24,25^ Lack of knowledge and reliable information about the benefits of MMC discouraged men as they claimed no benefits if they were already HIV-positive; had good hygiene; and were already practising other HIV-prevention methods such as the ‘Abstinence’ ‘Be faithful’ ‘Condomise’ (ABC) method.^48,50^ Furthermore, in previously non-circumcising communities in South Africa, men did not know that the MMC service was free at the local clinic or hospital.^21^ Secondary to a lack of knowledge, myths tend to impede the utilisation of SRH services. For example, post-circumcision, men opted not to use condoms after getting circumcised, citing reasons such as the circumcised penis would tear a condom; or putting a condom on an exposed circumcised penis would cause pain.^27,39,41^ In some studies, men associated circumcision with infertility.^47,48,49^ Equally, men often confuse vasectomy with castration and wrongly associate it with loss of libido, decreased sexual activity, and loss of masculinity.^46,51^

#### Individual or personal factors

In this review, individual or personal factors are defined as behaviour and characteristics demonstrated by men which determined whether they utilised SRH services or not. Fear emerged as the dominant barrier among individuals as an obstacle to utilising SRH services. Among the studies focused on MMC, fear of post-operative complications such as pain, delayed recovery, infections, loss of morning erections, and the post-procedure abstinence period presented major barriers to MMC.^21,47,48,49^ Post-circumcision myths, such as the inability to sexually satisfy partners and decreased penile sensitivity on a circumcised penis, also dissuaded certain men.^29,30^ Men were reluctant to utilise HIV services, fearing the possibility of testing positive, hence death related to HIV complications because of self-knowledge of infidelity. Men also feared the stigma associated with HIV, the possibility of being blamed and rejected by significant others such as partners, family members, and friends, and the pressure from employees to quit their job after diagnosis.^25,26,36,37,42,43,54^ Condom-use-associated barriers included poor quality of condoms, embarrassment when buying condoms, and perceived low risk.^26,27,41^ In Nigeria, some men were reluctant to screen for PC because there was no family history, and they did not believe they were at risk.^38^

#### Sociocultural and religious factors

Most cultural and religious practises consider discussing sexual matters taboo. Therefore, the sensitivity of discussing SRH issues has hindered many men from accessing SRH services.^42,47^ Factors associated with culture, such as the threat to masculinity, deterred men from undergoing MMC. For example, non-circumcising communities presumed circumcision to be an alien culture or part of a foreign religion.^29,30^ Some men referred to undergoing MMC as tampering with God’s creation.^45,50^

#### Socioeconomic factors

Sexual and reproductive health service utilisation was hindered by their perceived high costs and related products such as vasectomy and condoms (particularly in rural areas).^27,35^ In addition, the lack of access to condoms contributed to low utilisation.^39^ Taking time off work and losing income while, for example, waiting for the wound to heal post-MMC deterred some men from undergoing MMC.^44,47^ Furthermore, travelling costs to healthcare establishments also emerged as a significant deterrent as men lacked the money for transport.^25,27^ Health service utilisation is also compromised by high human geographic mobility as men constantly relocate from rural to urban areas in search of employment. Consequent to high mobility, negative interactions with healthcare providers, language barriers, and missed appointments may discourage health-seeking.^58^ This internal migration is historically coupled with the Apartheid system that restricted black South Africans from permanently settling in the urban areas where they were recruited to work in mines.^59^ Migration can hinder adherence to and continuity of healthcare for men who already had contact with health services.^58^

#### Geographical factors

Health facilities are ordinarily concentrated in urban rather than rural areas; however, most regions in LMICs are rural.^60^ Consequently, long distances and poor transport (especially in rural areas) to the health facility are barriers to accessing and utilising SRH services.^30,31,45,52^

### Facilitators to men’s utilisation of sexual and reproductive health services

Factors facilitating men’s utilisation of SRH services are summarised into health service system, knowledge, individual and/or personal issues, and socioeconomic factors.

#### Health service system (physical accessibility, availability, accessibility, affordability)

Health workers’ welcoming and friendly attitudes and respect for men’s privacy and confidentiality motivated men to access and utilise SRH services.^25,40,53^ Access to the right information about SRH services via advertisements such as pamphlets and radio or television programmes^25^ and support from healthcare providers also played a vital role in encouraging SRH service use by men.^23,43^

#### Knowledge

The benefits of undergoing MMC and using condoms, such as protection against diseases and improved hygiene, motivated many men to undergo the procedure.^21,30,42,47,51,55^ Men who knew that vasectomy was a low-risk procedure, with few complications and side effects and unlikely fail, were motivated to undergo the procedure to limit family size.^46,51^

#### Individual or personal factors

In some studies, men were motivated to perform MMC as they believed women were better satisfied sexually after circumcision. Wearing condoms was much easier after the foreskin had been removed.^22,47,42^ Furthermore, personal gain or prestige from research activities, such as free medical care, financial incentives, and a sense of responsibility from research, motivated men to utilise SRH services.^21,23,25,42^ Role modelling positive HIV status disclosure and adherence to antiretroviral therapy (ART) motivated men to engage in HIV treatment initiatives.^25,43^ Furthermore, an individual’s desire to limit family size encouraged some men to undergo a vasectomy as it was perceived as a permanent method with a low risk of complications, thus limiting the side effects of other female-controlled hormonal methods.^46,51^

#### Socioeconomic factors

Although vasectomy may be free in some LMICs, getting an appointment for the procedure can be a long process. However, men who can secure an appointment for the procedure or those who can afford to pay to counteract financial difficulties decided to undergo a vasectomy to limit family size and reduce expenses. Therefore, the dire socioeconomic state can be a motivating factor in undergoing vasectomy.^35,36^

## Discussion

This narrative review aimed to establish the factors influencing whether men do or do not utilise SRH services. In this review, only studies conducted in LMICs were included for synthesis. Identified studies were conducted mostly in the African regions (Southern, Eastern, and Western) and only 10% were conducted in South Asia. Although studies were conducted in either rural or urban areas (or in both rural and urban areas), findings in these settings remained comparable. Research indicates less focus on SRH issues such as PC screening, ED, and vasectomy services. The assumption may be less utilisation of these services because of high costs, as these SRH issues may be freely available only in some government health establishments in LMICs. Research in L and MIC is often conducted among unemployed and rural-based communities where men may not be able to afford such services, and sometimes they are against their culture and religious beliefs.^44,50^ However, further research is required to identify the reasons behind this. Furthermore, governments should make men’s SRH services affordable and accessible.

Fear plays a vital role in inhibiting men’s use of SRH services. The most common barriers related to personal factors included fear, embarrassment, and insecurity related to self-esteem and reputation. Men are discouraged from utilising SRH services such as MMC, vasectomy services, FP, STI and/or HIV, and PC screening.^21,22,23,24,25,26,27,28,29,30,33,34,35,36,42,43,44,45^ Many men find it embarrassing to go to SRH services and consider it a very negative experience when they are seen, ridiculed, and disrespected by people known to them.^39,40,41,46,47,48,49,50,51,52,53,54,55^ These negative experiences fundamentally discourage SRH service utilisation by men.

The under-utilisation of SRH services is associated with a range of cultural barriers. For example, cultural and religious backgrounds perceive discussing sexual matters as taboo and deter men’s utilisation of SRH services.^29,33^ The threat to masculinity, especially secondary to misconceptions and myths, significantly deters men from utilising SRH services such as MMC or vasectomy. Men are often concerned about the inability to produce more children, the loss of manhood, and infertility.^31,35,47^ Despite the availability of services, poor utilisation of SRH remains associated with limited knowledge of the various available forms of SRH services. Lack of knowledge or awareness of SRH services such as vasectomies, screening for PC, STI and/or HIV testing, MMC, centres for counselling, and information provision contribute to men’s low utilisation of SRH services.^25,30,31,32,33,34,35,36,37,38,39,40,41,42,43,44,45^ Despite the many educational campaigns provided on various SRH services in the past, governments still need to roll out sustained ongoing campaigns at schools, workplaces, communities, and during weekends.

Certain studies reveal financial constraints as fundamental inhibitors in accessing and utilising SRH services such as vasectomies, PC services, and MMC. Although most SRH services are free in some LMICs, some men, especially from rural-based settings, allude to high cost as a hindrance to accessing SRH services.^21,34,35^ Such difficulties in accessing SRH services may be due to unemployment. In remote areas, several health facilities are far from the communities. Consequently, people in rural-based communities must walk long distances because of a lack of money and transport.^30,33,60^ Moreover, in some health establishments, healthcare workers’ conduct significantly influences whether men do or do not utilise SRH services. Men’s utilisation of SRH services is predominantly deterred by bad health workers’ attitudes and lack of privacy and confidentiality.^22,32,33,44,45^

Amid studies that investigated enablers of SRH service utilisation, virility motivated men to undergo MMC. Post-MMC, some men claim to be stronger sexually and that wearing condoms is much easier.^47,48,49^ Knowledge is also instrumental in encouraging SRH service utilisation. Men who understand the benefits of MMC are motivated to perform the procedure.^21,30,44^ It is imperative also to note that men need the constant provision of SRH information, especially about the benefits and existence of various SRH services. In addition, professional and better treatment from healthcare workers encourages SRH service utilisation.^25^

Despite the facilitating factors, most studies revealed that barriers to SRH service utilisation outweighed and require urgent attention. Some studies identified low utilisation of SRH services despite being available at low cost or no cost in some settings.^20^ Low SRH utilisation contributes to high morbidity and mortality among men and indirect mortality among women. Low SRH service utilisation, such as MMC, indirectly contributes to high HIV infection rates. In South Africa, almost 20% of adults aged 15–49 years are estimated to be HIV positive, while 8.2 million people live with HIV.^60^ HIV infection rates and low uptake of MMC remain major concerns in LMICs.

Nevertheless, multiple studies suggest that almost 80% coverage of MC would be necessary to impact HIV significantly and decrease incidence by at least 35%.^21,48^ Moreover, a positive trend in the uptake of HIV testing is well-documented. Meanwhile, a significant number of people still require testing.^25^ The effects of the HIV pandemic have created a shortage of human resources while plunging most countries into financial crises and knocking down national treatment implementation plans.^24^ High HIV infection rates in the general population indicate the need for various effective prevention measures, such as high uptake of circumcision, to reduce the incidence of HIV infections.^22^

Furthermore, limited knowledge, low-risk perceptions, unknown HIV statuses, and the high rates of STDs in some LMICs illustrate less condom use.^19^ High sexually transmitted infections among men may also be linked to STD screening programmes that unilaterally focus on women during the reproductive period. Men are less likely to seek or receive regular screening for SRH care, and most countries lack STD screening programmes to support regular screening in men.^50,52^ A multi-country HIV study conducted in sub-Saharan Africa found that men were less likely to take advantage of the programme and test for HIV compared to women. In some instances, where an equal proportion of men and women used HIV testing services, men would only get tested for HIV after becoming severely ill.^43^ The gender gap in health-seeking behaviour among men has been associated with the masculinity factor. Masculinity is a patriarchal, culturally promoted status quo held by men.^23^

Secondary to cultural beliefs, political barriers, and economic challenges, men still highly influence fertility and contraceptive use in many societies in LMICs.^33^ Despite excellent health policies to improve SRH service utilisation, many LMICs still face low SRH service uptake leading to the inability to exercise fertility preferences. Consequently, fertility remains high despite substantial FP policies that allow modern contraceptive use without consent from a partner or parent, especially in communities experiencing poor socioeconomic status.^35,51^ Therefore, identifying barriers to contraceptive uptake is warranted, especially in areas with high fertility. In LMICs such as Uganda, persistent high fertility contributes to high maternal morbidity and mortality, and a rapidly growing population.

Moreover, less contraceptive use, further increases unintended pregnancies resulting in high maternal deaths.^54^ Consequently, public resources are strained, and opportunities for economic development are hampered.^45^ Contraceptive uptake can avert millions of preventable deaths, including infant and maternal deaths.

Vasectomy remains one of the least known and least used FP methods in LMICs. The prevalence is less than 1% in LMICs compared to more than 12% in developed countries.^46^ Henceforth it is considered one of the most reliable FP methods currently available, with very low post-vasectomy pregnancy rates.^35^ Myths and limited knowledge hinder vasectomy acceptability and its uptake in LMICs. Research on vasectomy has shown that orienting FP services towards men and increasing their knowledge of the method through various media outlets may improve its uptake.^46^

The utilisation of SRH services by men is influenced by a complex set of factors related to SRH knowledge; personal; sociocultural norms and beliefs; political will; socioeconomic factors, availability and accessibility of services; quality of the services.^55^ The analysis also reveals that men generally are reluctant to seek and engage with SRH services for anything other than severe illness.^40,53,56^ Other studies indicate that poor health-seeking behaviour among men requires urgent attention from policymakers. Besides experiencing barriers to care at personal, social, and cultural levels, one possible explanation for men’s low levels of SRH care is a need for greater interest in talking with their healthcare provider about these issues.^4^

Sexual and reproductive health information and services are provided through different forms of media, local health pharmacists, public health practitioners, doctors, nurses, and community health workers. However, HIV infection rates, fertility, and unintended pregnancies indicate less frequently used SRH services.^41^ This abysmal SRH information provision is predominant in urban areas while remaining poor in rural communities.^40,53^

This narrative review of the literature revealed a limited number of studies focusing on factors influencing whether male use of SRH services in LMICs. Given the lack of focus on men’s SRH by international programmes such as SDGs 2030 and FP2020,^12,18^ more studies on men must be conducted, especially in LMICs. Research focusing on men may bring about evidence-based knowledge that will compel policymakers to utilise it when planning to provide SRH services for men. When factors hindering and facilitating men’s SRH service utilisation are known, understood, and dealt with comprehensively and locally, men’s utilisation of SRH services could improve. Subsequently, high morbidity and mortality among men may be averted. The global community must answer the critical question – ‘why do we ignore gender inequity when it impacts the health of boys and men?’. Poor leadership and management are fundamentally linked to failing health systems.^59^ Therefore, attending to the systemic health barriers is essential to ensure sexual and reproductive justice for all.^27^

**FIGURE 2:**
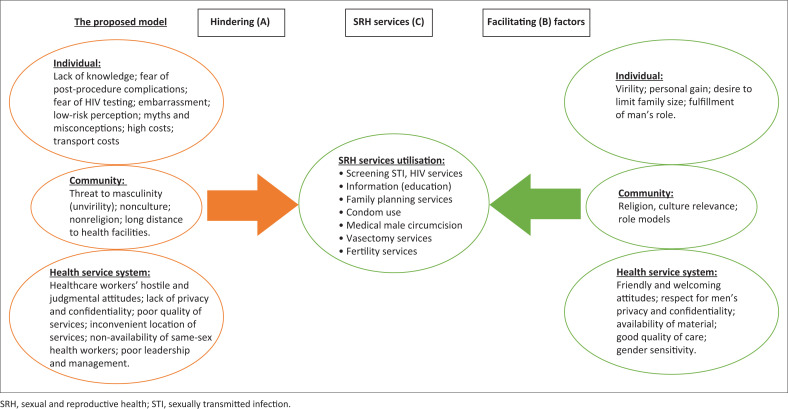
A conceptual model for understanding men’s engagement with sexual and reproductive health services.

Drawing from the findings of this analysis, we constructed the conceptual model that illustrates factors hindering or facilitating men’s SRH service utilisation (Figure 2). In the model, key barriers to and motivators for SRH service utilisation are listed in boxes A and B, respectively, whereas SRH services utilised are listed in box C. This conceptual model can be used by policymakers, service administrators, and service providers to learn about men’s barriers and facilitators to SRH service utilisation in the context of LMICs.

## Limitations of the review

This review was confine to LMIC studies only. Therefore, findings may not be generalisable to other settings. The narrative review’s authors also restricted the criteria to include studies published in English because of insufficient resources for translation. English studies may limit how the review’s findings can be transferable to all LMICs. We did not conduct additional study quality assessments or remove studies based on the risk of bias, given that our goal was to describe the relevant studies identified. The predominant focus on VMMC from many studies may result in biased reporting. Narrative reviews often do not meet important criteria to help mitigate bias because they frequently lack explicit criteria for article selection. The paucity of studies focusing on men’s barriers and facilitators to SRH service utilisation may reduce the complexity of the argument. We also included articles that investigated both men and women and those aged 15 years and older, which may have included adolescents. However, authors considered results where authors explicitly separated men’s and women’s results by subheadings. Authors, however, acknowledge that this may introduce bias in the analysis of the results.

## Implications

This review consolidated knowledge about barriers and facilitators influencing men to utilise SRH services. While reviews conducted in the past decades have identified barriers and facilitators to SRH services, our narrative review is the first to comprehensively focus on all SRH services or issues, and men exclusively. The findings from this review have implications for clinical practise and policy.

## Conclusion and recommendations

Despite the need for SRH utilisation by men, there is little evidence of successful interventions. Consequently, low SRH utilisation has become a major concern regarding men’s health. Men’s SRH service utilisation must be investigated comprehensively to comprehend men’s problems fully. None of the identified studies comprehensively investigated SRH issues. There is also a need for studies investigating vasectomies and their influence on fertility.

There is a need to explore the utilisation of SRH services among older men as most studies predominantly focus on adolescents and young men, as well as on barriers rather than facilitators to SRH service utilisation. Although SRH includes mental health issues, little is known about mental health issues related to sexual health. The same applies to the impact of physical disabilities and chronic illnesses on sexual well-being. Further research may be warranted in this regard. Governments must increase the awareness and education of the public to fight against myths and misconceptions linked to the utilisation of SRH services and improve healthcare providers’ capacity to engage men better. Furthermost, SRH policymakers and programme managers must avoid frequent changes in SRH prevention programmes as that leads to confusion, despair, and distrust in the health system while discouraging men from engaging with SRH services.^37,48,54^

## References

[1] Jobson M. Structure of the health system in South Africa. South Africa Khulumani Support Group: Johannesburg; 2015.

[2] Starrs AM, Ezeh AC, Barker G, et al. Accelerate progress – Sexual and reproductive health and rights for all: Report of the Guttmacher – Lancet Commission. Lancet. 2018;391(10140):2642–2692. 10.1016/S0140-6736(18)30293-9 29753597

[3] De Silva VP. World book of family medicine: Sexual and reproductive health in primary care: Where do we go from here? vol. 26. Wonca Europe: Iberoamericana Edition: Portugal; 2016, p. 1–4.

[4] Same RV, Bell DL, Rosenthal SL, Marcell AV. Sexual and reproductive health care: Adolescent and adult men’s willingness to talk and preferred approach. Am J Prev Med. 2014;47(2):175–181. 10.1016/j.amepre.2014.03.009 24951042

[5] Department of Health (DOH). Sexual and reproductive health and rights: Fulfilling our commitments 2011–2021 and beyond [homepage on the Internet]. 2011 [cited 2020 Jun 21]. Available from: http://www.agenda.org.za

[6] World Health Organization. Sexual health and its linkages to reproductive health: An operational approach [homepage on the Internet]. 2017 [cited 2020 Jul 16]. Available from: https://www.who.int

[7] World Health Organization. Developing sexual health programmes: A framework for action [homepage on the Internet]. World Health Organization; 2010 [cited 2021 May 17]. Available from: https://www.gov.uk

[8] Forrest KA. Men’s reproductive and sexual health. J Am College Health. 2001;49(6):253–266. 10.1080/07448480109596312 11413943

[9] Ghebreyesus TA, Kanem N. Defining sexual and reproductive health and rights for all. Lancet. 2018;391(10140):2583–2585. 10.1016/S0140-6736(18)30901-2 29753598

[10] Shand T, Zamir J, Marcell AV, Perlson S. Global sexual and reproductive health service package for men and adolescent boys [homepage on the Internet]. London: IPPF; 2017 [cited 2020 Aug 15]. Available from: https://www.unfpa.org

[11] Griffin S. Universal access to sexual and reproductive health services. Eldis Health Key Issues [homepage on the Internet]. 2006 [cited 2020 Jul 22]. Available from: https://assets.publishing.service.gov.uks

[12] Green A. Man to man, sexual health needs are better met at male-only clinics. Cape Town: Sonke Gender Justice; 2015.

[13] Health Communication Capacity Collaborative (HC3). Guide for promoting sexual and reproductive health products and services for men [homepage on the Internet]. Baltimore, MD: Johns Hopkins Center for Communication Programs (CCP); 2017 [cited 2020 Sept 12]. Available from: https://healthcommcapacity.org

[14] Baker P, Shand T. Men’s health: Time for a new approach to policy and practice? J Glob Health [serial online]. 2017 [cited 2020 Jul 26];7(1). Available from: https://www.ncbi.nlm.nih.gov/pmc/articles/PMC5344012 10.7189/jogh.07.010306 PMC5344012 28400949

[15] EngenderHealth. Men’s reproductive health curriculum: Introduction to men’s reproductive health services [homepage on the Internet]. 2008 [cited 2021 Jun 13]. Available from: http://www.medbox.org

[16] Mnyulwa B. One man can’s first clinic in Gugulethu: Our pride [homepage on the Internet]. Cape Town: Sonke Gender Justice; 2011 [cited 2020 Oct 23]. Available from: https://genderjustice.org.za

[17] Kalmuss D, Tatum C. Patterns of men’s use of sexual and reproductive health services. Perspect Sex Reprod Health. 2007;39(2):74–81. 10.1363/3907407 17565620

[18] Sonke Gender Justice. Men for change, health for all: A policy discussion paper on men, health and gender equity [homepage on the Internet]. Cape Town: Sonke Gender Justice Network; 2008 [cited 2020 Jun 17]. Available from: https://genderjustice.org.za

[19] Zuma T. mHealth for men: Development of a home-based intervention to test and start (HITS) to support HIV testing and early linkage to care amongst men in rural KwaZulu-Natal, South Africa. Durban: Africa Health Research Institute; 2018.

[20] Hoffman JR, Arendse KD, Larbi C, Johnson N, Vivian LM. Perceptions and knowledge of voluntary medical male circumcision for HIV prevention in traditionally non-circumcising communities in South Africa. Glob Public Health. 2015;10(5–6):692–707. 10.1080/17441692.2015.1014825 25727250

[21] Humphries H, Van Rooyen H, Knight L, Barnabas R, Celum C. ‘If you are circumcised, you are the best’: Understandings and perceptions of voluntary medical male circumcision among men from KwaZulu-Natal, South Africa. Cult Health Sex. 2015;17(7):920–931. 10.1080/13691058.2014.992045 25567140 PMC4470729

[22] Zissette S, Watt MH, Prose NS, Mntambo N, Moshabela M. ‘If you don’t take a stand for your life, who will help you?’: Men’s engagement in HIV care in Kwazulu-Natal, South Africa. Psychol Men Masc. 2016;17(3):265. 10.1037/men0000025 27616937 PMC5012535

[23] Khan R, Yassi A, Engelbrecht MC, Nophale L, Van Rensburg AJ, Spiegel J. Barriers to HIV counseling and testing uptake by health workers in three public hospitals in Free State Province, South Africa. AIDS Care. 2015;27(2):198–205. 10.1080/09540121.2014.951308 25174842

[24] Mwisongo A, Mohlabane N, Tutshana B, Peltzer K. Barriers and facilitators associated with HIV testing uptake in South African health facilities offering HIV counselling and testing. Health SA Gesondheid. 2016;21(1):86–95. 10.1016/j.hsag.2015.11.001

[25] Stern E, Cooper D, Rau A. Sexual and reproductive health perceptions and practices as revealed in the sexual history narratives of South African men living in a time of HIV/AIDS. SAHARA J. 2014;11(1):233–244. 10.1080/17290376.2014.985701 25495581 PMC4272193

[26] Morison T, Lynch I. Use and perceptions of public sexual and reproductive health services: A quantitative situational analysis in OR Tambo and Gert Sibande districts; Durban: Aids Foundation of South Africa; 2016.

[27] Chikovore J, Gillespie N, McGrath N, Orne-Gliemann J, Zuma T. Men, masculinity, and engagement with treatment as prevention in KwaZulu-Natal, South Africa. AIDS Care. 2016;28(sup3):74–82. 10.1080/09540121.2016.1178953 27421054 PMC5096677

[28] Evens E, Lanham M, Hart C, Loolpapit M, Oguma I, Obiero W. Identifying and addressing barriers to uptake of voluntary medical male circumcision in Nyanza, Kenya among men 18–35: A qualitative study. PLoS One. 2014;9(6):e98221. 10.1371/journal.pone.0098221 24901226 PMC4047024

[29] Herman-Roloff A, Otieno N, Agot K, Ndinya-Achola J, Bailey RC. Acceptability of medical male circumcision among uncircumcised men in Kenya one year after the launch of the national male circumcision program. PLoS One. 2011;6(5):e19814. 10.1371/journal.pone.0019814 21603622 PMC3095626

[30] Withers M, Dworkin SL, Onono M, et al. Men’s perspectives on their role in family planning in Nyanza Province, Kenya. Stud Fam Plann. 2015;46(2):201–215. 10.1111/j.1728-4465.2015.00024.x 26059990

[31] Godia PM, Olenja JM, Lavussa JA, Quinney D, Hofman JJ, Van Den Broek N. Sexual reproductive health service provision to young people in Kenya; health service providers’ experiences. BMC Health Serv Res. 2013;13(1):1–3. 10.1186/1472-6963-13-476 24229365 PMC4225671

[32] Sunnu E, Adatara P, Opare FY, Kuug A, Nyande F. Factors influencing the utilisation of family planning contraceptives among men and women in the Ho Municipality of Ghana. Int J Health Sci Res 2016;6:204.

[33] Leblanc NM, Andes KL. An exploration of men’s knowledge, attitudes, and perceptions of HIV, HIV risk, and willingness to test for HIV in Yendi District, Northern Ghana. J Assoc Nurses AIDS Care. 2015;26(3):281–295. 10.1016/j.jana.2014.09.006 25456835

[34] Adongo PB, Tapsoba P, Phillips JF, et al. ‘If you do vasectomy and come back here weak, I will divorce you’: A qualitative study of community perceptions about vasectomy in Southern Ghana. BMC Int Health Hum Rights. 2014;14(1):1–8. 10.1186/1472-698X-14-16 PMC4019590 24885663

[35] Thomas KA. HIV/AIDS voluntary counselling and testing (VCT): Perspectives of rural youths in Oyo State, Nigeria. Int J Agric Econ Rural Dev. 2015;7(1):52–58.

[36] Jones OO. Factors affecting the uptake of voluntary counselling and testing among youth in rural Nigeria. Lethbridge: University of Lethbridge; 2017.

[37] Hassan R. Knowledge, perception, risk factors and utilization of prostate cancer screening services among male staff of the University College Hospital [Doctoral dissertation]. Ibadan: The African Digital Health Library (ADHL); 2015.

[38] Tamang L, Raynes-Greenow C, McGeechan K, Black KI. Knowledge, experience, and utilisation of sexual and reproductive health services amongst Nepalese youth living in the Kathmandu Valley. Sex Reprod Healthc. 2017;11:25–30. 10.1016/j.srhc.2016.09.002 28159124

[39] Gautam P, Soomro MH, Sapkota S, Gautam KR, Kasaju A. Barriers to utilization of sexual health services among young people in district dang Nepal: A qualitative study. J Med. 2018;19(2):79–83. 10.3329/jom.v19i2.37224

[40] Regmi PR, Van Teijlingen E, Simkhada P, Acharya DR. Barriers to sexual health services for young people in Nepal. J Health Popul Nutr. 2010;28(6):619. 10.3329/jhpn.v28i6.6611 21261208 PMC2995031

[41] Hatzold K, Mavhu W, Jasi P, et al. Barriers and motivators to voluntary medical male circumcision uptake among different age groups of men in Zimbabwe: Results from a mixed methods study. PLoS One. 2014;9(5):e85051. 10.1371/journal.pone.0085051 24802746 PMC4011705

[42] Skovdal M, Campbell C, Madanhire C, et al. Masculinity as a barrier to men’s use of HIV services in Zimbabwe. Glob Health 2011;7(1):13. 10.1186/1744-8603-7-13 PMC3107786 21575149

[43] Ssekubugu R, Leontsini E, Wawer MJ, et al. Contextual barriers and motivators to adult male medical circumcision in Rakai, Uganda. Qual Health Res. 2013;23(6):795–804. 10.1177/1049732313482189 23515302

[44] Nalwadda G, Mirembe F, Byamugisha J, Faxelid E. Persistent high fertility in Uganda: Young people recount obstacles and enabling factors to use of contraceptives. BMC Public Health. 2010;10(1):1–3. 10.1186/1471-2458-10-530 20813069 PMC2940919

[45] Shattuck D, Wesson J, Nsengiyumva T, et al. Who chooses vasectomy in Rwanda? Survey data from couples who chose vasectomy, 2010–2012. Contraception. 2014;89(6):564–571. 10.1016/j.contraception.2014.02.003 24630244

[46] Adams A, Moyer E. Sex is never the same: Men’s perspectives on refusing circumcision from an in-depth qualitative study in Kwaluseni, Swaziland. Glob Public Health. 2015;10(5–6):721–738. 10.1080/17441692.2015.1004356 25654269

[47] Kelly A, Kupul M, Fitzgerald L, et al. ‘Now we are in a different time; various bad diseases have come.’ Understanding men’s acceptability of male circumcision for HIV prevention in a moderate prevalence setting. BMC Public Health. 2012;12(1):1–3. 10.1186/1471-2458-12-67 22264256 PMC3298502

[48] Skolnik L, Tsui S, Ashengo TA, Kikaya V, Lukobo-Durrell M. A cross-sectional study describing motivations and barriers to voluntary medical male circumcision in Lesotho. BMC Public Health. 2014;14(1):1. 10.1186/1471-2458-14-1119 25359662 PMC4287583

[49] Yabeny TE. Assessing condom use among Navajo Men in the Southwest [Doctoral dissertation]. Walden University; 2018.

[50] Parcon CR. Men, family planning and contraceptive use in Western Visayas; Bingawan: Xytron Philippines Incorporated; 2010.

[51] Muntean N, Kereta W, Mitchell KR. Addressing the sexual and reproductive health needs of young people in Ethiopia: An analysis of the current situation. Afr J Reprod Health. 2015;19(3):87–99. 26897917

[52] Oraby DM. Sexual and reproductive health among young people in Egypt: The role and contribution of youth-friendly services. Sex Educ. 2013;13(4):470–477. 10.1080/14681811.2012.756810

[53] Thin Zaw PP, Liabsuetrakul T, Htay TT, McNeil E. Equity of access to reproductive health services among youths in resource-limited suburban communities of Mandalay City, Myanmar. BMC Health Serv Res. 2012;12(1):1–2. 10.1186/1472-6963-12-458 23241510 PMC3546958

[54] Thongmixay S, Essink DR, Greeuw TD, Vongxay V, Sychareun V, Broerse JE. Perceived barriers in accessing sexual and reproductive health services for youth in Lao People’s Democratic Republic. PLoS One. 2019;14(10):e0218296. 10.1371/journal.pone.0218296 31661486 PMC6818758

[55] Nyalela M, Dlungwane T, Taylor M, Nkwanyana N. Health seeking and sexual behaviour of men presenting with sexually transmitted infections in two primary health care clinics in Durban. S Afr J Infect Dis. 2018;33(5):1–6. 10.1080/23120053.2018.1520480

[56] Tsadik M, Lam L, Hadush Z. Delayed health care seeking is high among patients presenting with sexually transmitted infections in HIV hotspot areas, Gambella town, Ethiopia. HIV/AIDS (Auckland, NZ). 2019;11:201–209. 10.2147/HIV.S210977 PMC6724613 31564990

[57] Ginsburg C, Collinson MA, Gómez-Olivé FX, et al. Internal migration and health in South Africa: Determinants of healthcare utilisation in a young adult cohort. BMC Public Health. 2021;21(1):1–5. 10.1186/s12889-021-10590-6 33743663 PMC7981972

[58] Coovadia H, Jewkes R, Barron P, Sanders D, McIntyre D. The health and health system of South Africa: Historical roots of current public health challenges. Lancet. 2009;374(9692):817–834. 10.1016/S0140-6736(09)60951-X 19709728

[59] Spasojevic N, Vasilj I, Hrabac B, Celik D. Rural–urban differences in health care quality assessment. Mater Socio Med. 2015;27(6):409. 10.5455/msm.2015.27.409-411 PMC4753384 26937222

[60] Maluleke R. Mid-year population estimates. Pretoria: Statistics South Africa; 2021.

